# Proteomic Signatures of Vemurafenib Resistance in Canine Urothelial Carcinoma Harboring the BRAF^V595E^ Mutation

**DOI:** 10.1021/acs.jproteome.6c00246

**Published:** 2026-06-26

**Authors:** Rodrigo Mohallem, Deepika Dhawan, Alexander W. Enstrom, Deborah W. Knapp, Uma K. Aryal

**Affiliations:** 1 Department of Comparative Pathobiology, College of Veterinary Medicine, 311308 Purdue University, West Lafayette, Indiana 47907, United States; 2 Department of Veterinary Clinical Sciences, College of Veterinary Medicine, 311308 Purdue University, West Lafayette, Indiana 47907, United States; 3 Purdue University Institute for Cancer Research, West Lafayette, Indiana 47907, United States

**Keywords:** anticancer therapy, canine, invasive urothelial Carcinoma, mass spectrometry, mutated BRAF, proteomics, vemurafenib

## Abstract

Naturally occurring canine invasive urothelial carcinoma (InvUC) often harbors a BRAF^V595E^ mutation, analogous to human BRAF^V600E^ found across multiple cancer types, and has been used to investigate the effects of BRAF-targeted therapy. To investigate proteomic and phosphoproteomic changes during therapy, we analyzed 42 samples from 14 dogs with BRAF^V595E^-positive InvUC treated with vemurafenib. Tumors were collected via cystoscopy before treatment (Pre-Vem), 1 month into treatment (Vem-1-month), and at the time of progressive disease (Vem-PD). We observed consistent early suppression of proteins and phosphosites in tumors after 1 month of vemurafenib treatment in dogs that responded to the treatment, followed by their rebound at the time of progressive disease, highlighting early tumor suppressive events that are later reversed as tumors adapt and develop resistance. Key pathways affected included "positive regulation of telomerase assembly and localization to Cajal body", "positive regulation of protein localization to telomere", and "positive regulation of protein localization to Cajal body". Additionally, consistent "Rho GTPase signaling" changes were observed across both proteomic and phosphoproteomic analyses, underscoring the functional reactivation of this pathway in resistant tumors. In summary, these findings reveal molecular signatures of early response and acquired resistance to vemurafenib and offer a valuable resource for future investigation of BRAF-targeted therapy.

## Graphical abstract

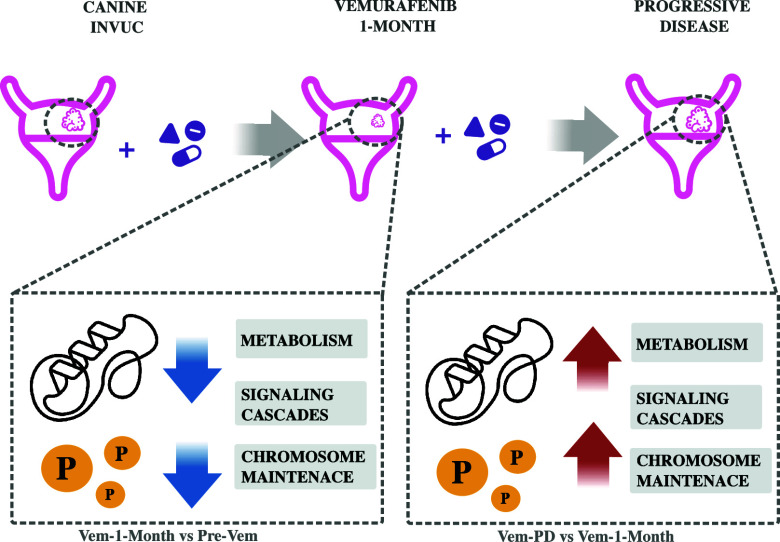

## Introduction

Cancer is the second most common cause of death worldwide, and the number of cases is expected to rise to 21 million by 2030.^1^ Cancer cells accumulate hundreds of mutations that may be inherited or develop over time through a combination of genetic and environmental factors, including exposure to specific chemicals or radiation. Diverse mutations result in tumor heterogeneity, which is a key driver of poor drug response, a major challenge in cancer therapy.^2^ Along with surgery and radiation therapy, cancer treatment often involves a combination of chemotherapy, targeted drug therapy, and immunotherapy. A better understanding of the mechanisms that drive the antitumor effects and resistance to cancer drugs is necessary to improve cancer therapies.

Dogs with naturally occurring cancer are continuing to gain attention in research to improve cancer therapies for humans.^3^ Applying genetic and computational tools, researchers have identified hundreds of gene mutations underlying many diseases.^4^ Almost half of the 450 known hereditary diseases in dogs have clinical similarities to corresponding human diseases.^5^ Dogs are known to develop multiple forms of cancer that are similar to those in humans, and these cancers are often treated with the same drugs or similar drugs used for human patients.^6^ Sequenced genomes^7^ and numerous genomic and postgenomic studies to identify genetic mutations that are present in canine and human cancer^8,9^ are justifying the use of companion dogs as models in the search for new anticancer drug targets. While traditional mouse and human tumor xenograft models have advanced our molecular understanding of cancer and the development of anticancer drugs, the results of studies in these models do not necessarily correlate with findings in humans.^10,11^ This can be attributed to the higher complexities of cancer and substantial differences between and within patients in disease pathways, immune responses, and a tumor microenvironment that exist in human cancer but not in experimental tumor models.^12–14^ Pet dogs, in contrast, develop diverse and complex forms of cancer,^15^ and naturally occurring cancers in pet dogs offer complementary alternative animal models to inform rational drug development in human cancers.^7,9,16,17^

One of the cancer types studied for translational research is naturally occurring invasive urinary bladder cancer in companion dogs, specifically invasive urothelial carcinoma (InvUC), also known as transitional cell carcinoma, which is the most common form of bladder cancer in dogs.^18,19^ This high-grade and heterogeneous bladder carcinoma closely mimics muscle invasive bladder cancer in humans in clinical and pathologic features, local invasion, distant metastasis in 50–60% of cases, and in harboring molecular subtypes similar to those in human InvUC.^18,20,21^ Interestingly, more than 80% of canine InvUCs have a mutation in the proto-oncogene serine/threonine kinase BRAF (BRAF^V595E^), the canine homologue to human BRAF^V600E^.^18,22,23^ In humans, BRAF mutations are common across cancer types but are limited to the most aggressive bladder cancer cases.^24^ InvUC in dogs offers a translationally relevant model to investigate potential drugs and treatment strategies for humans.^15^ Thus, characterizing InvUC in dogs and their response to treatment is highly relevant, particularly due to similarities to human medical care, comparable host and organ size, and cohabitation leading to similar environmental exposures.^5,25^

In our earlier canine phase I/II clinical trial, the BRAF inhibitor, vemurafenib, had a very good antitumor activity in pet dogs with InvUC^26^ harboring the BRAF^V595E^ mutation. In fact, only two dogs had inherent resistance to vemurafenib, and the remaining dogs had partial remission or stable disease. Vemurafenib inhibits kinase activity and signaling in the MAPK pathway, thus blocking malignant cell proliferation.^27–30^ The BRAF^V600E^ mutation is detected in 8% of all human cancers and in up to 24% of aggressive InvUC in human patients. While vemurafenib has been effective against advanced cancers in humans especially in melanoma,^31,32^ inherent and acquired drug resistance remain major problems.^33–36^ The resistance mechanisms can involve MAPK pathway reactivation, the activation of alternative signaling pathways, and immunologic effects,^37–39^ but overall remain poorly understood,^32,33^ highlighting the need for further investigation. Acquired drug resistance is especially common and is often lethal, and uncovering the mechanisms involved in this failure is a focus of this report.

The purpose of this study was to determine mechanisms underlying the tumor response to BRAF-targeted therapy and, more specifically, the mechanisms involved in initial tumor control followed by acquired drug resistance. Advanced proteomic and phosphoproteomic profiling was applied to 42 InvUC tissue samples collected via cystoscopy from 14 companion dogs with InvUC before and during vemurafenib treatment. This study complements our previous RNA-seq study,^26^ providing additional understanding of molecular changes in InvUC tissues during the course of the treatment. Here, we focused our analysis on proteins and phosphorylated residues that had significant variations that were correlated with acquired vemurafenib resistance. Our results indicate that tumor resistance to vemurafenib is linked to extensive metabolic reprogramming in tumor cells, including the upregulation of proteins involved in cell proliferation and the dysregulation of protein phosphorylation. These changes contribute to the reactivation of the Rho GTPase signaling and MAP kinase pathways. Overall, proteomic and phosphoproteomic analyses before and during vemurafenib treatment in dogs offer valuable insights into the molecular pathways driving InvUC tumor responses and reveal key mechanisms underlying drug resistance, thereby advancing our understanding of BRAF-targeted effects and resistance in cancer therapy.

## Materials and Methods

### Study Subjects and Ethics Statement

InvUC tissue samples banked from dogs in a phase I/II trial of vemurafenib were studied. Information from the trial including entry criteria, drug administration, clinical evaluation methods, maximum tolerated dose (MTD), clinical outcomes, group comparisons, and pharmacokinetic analysis has been published.^26^ The study was approved by the Institutional Animal Care and Use Committee of Purdue University (IACUC protocol no. 1111000124), complied with relevant national and institutional regulations, and was conducted and reported in accordance with the ARRIVE 2.0 guidelines. The trial was conducted in the Canine Bladder Cancer Clinic in the Purdue University Veterinary Hospital (PUVH) in dogs with InvUC harboring the BRAF^V595E^ mutation. All dog owners provided informed consent, and the dogs lived at home with their owners other than scheduled hospital visits for evaluation before and during treatment.

All dogs in the study had histopathologically confirmed lower urinary tract InvUC with BRAF^V595E^ mutation by analysis as described in ref 23. All tumor tissues were reviewed and graded by a single pathologist. Vemurafenib (Zelboraf, Genentech, 240 mg tablets) was administered orally with food, as a single agent. Tumor responses were defined as complete remission (CR: no residual cancer detected), partial remission (PR: ≥ 50% decrease in tumor volume and no new tumor lesions), stable disease (SD: < 50% change in tumor volume and no new tumor lesions), and progressive disease (PD: ≥ 50% increase in tumor volume or development of new lesions) as described previously.^20,26^

Cystoscopy was performed prior to vemurafenib treatment (Pre-Vem), after 1 month of vemurafenib (Vem-1-month), and, when possible, at cancer progression (Vem-PD). The dogs studied here had biopsies available from all three time points, received vemurafenib at or near the maximum tolerated dose (37.5 mg/kg) as allowed by tablet size, and had an initial tumor response of SD or PR. A total of 42 tumor biopsies from 14 dogs were subjected to global and phosphoproteomic analyses (Figure 1A). Raw LC-MS/MS data were processed with the MaxQuant and Perseus platforms.

**Figure 1:**
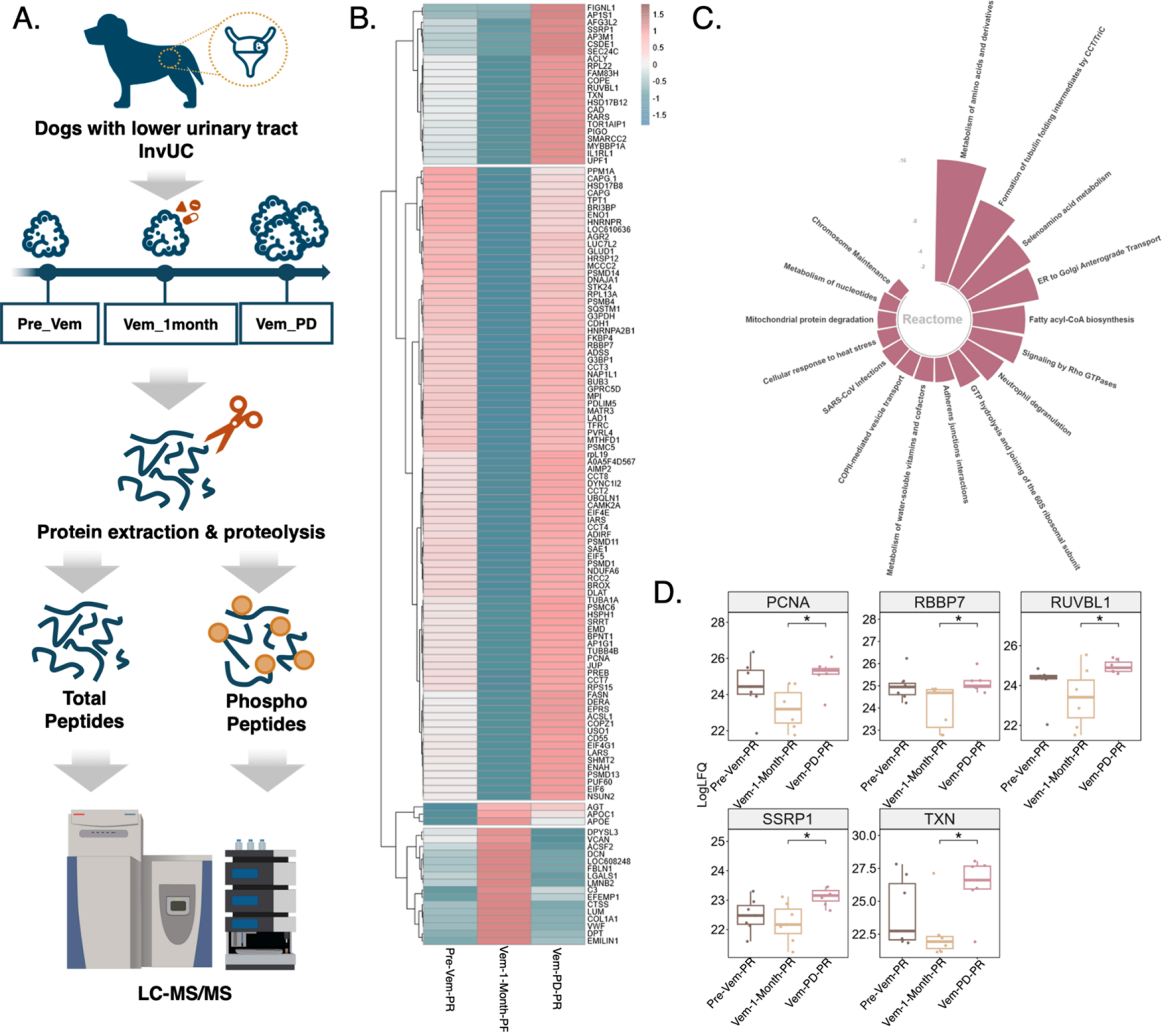
Global proteomics analysis of InvUC tissues in dogs treated with vemurafenib. (A) Proteomics and phosphoproteomic workflow. InvUC tissues were collected prior to vemurafenib treatment (Pre-Vem), 1 month after the start of vemurafenib treatment (Vem-1-month), and at the onset of progressive disease (Vem-PD). Collected tumor tissues were homogenized and processed for LC-MS/MS analysis. A subset of peptides was used for global analysis, and the remainder used for phosphopeptide enrichment prior to LC-MS/MS analysis. (B) Heatmap representation of proteins that were significantly different among the three different time points in dogs that had PR at the 1 month time point (ANOVA *p-*value <0.05). Red hue indicates positive *z*-score values, while blue hue indicates negative *z*-score values. Breaks in the heatmap represent the division between different clusters. (C) Reactome analysis of proteins significantly upregulated in Vem-PD relative to Vem-1-month in the dogs that achieved PR. Bar height indicates enrichments −log­(*P*-value). (D) Box plots for proteins mapped to the Reactome term "chromosome maintenance" (*p < 0.05; one-way ANOVA followed by Tukey HSD test).

### Proteomics and Phosphoproteomics Sample Preparation

Tumor biopsies were homogenized in a Precellys tissue homogenizer (Bertin Technologies, Rockville, MD, USA) following the protocol described previously.^26,40^ Protein concentration in the homogenate was measured by the bicinchoninic acid (BCA) assay. Five hundred (500) micrograms of protein (equivalent volume) of the homogenate was precipitated by adding four volumes of cold acetone overnight at −20 °C. Reduction, alkylation, and digestion were accomplished as described previously.^41^ Briefly, digestion was performed in a NEP2320 Barocycler (Pressure Biosciences, South Easton, MA, USA) using a high-pressure digestion system set at 50 °C, 60 cycles of 50 s at 20 kpsi, and 10 s at atmospheric pressure. Digested peptides were desalted using Pierce Peptides Desalting Spin Columns (Catalog No. 89851, Thermo Fisher Scientific, Waltham, MA) following the manufacturer’s protocol. These ready-to-use columns contain a polymer-based hydrophobic resin for removing salts and MS-interfering contaminants from peptide samples. Briefly, digested peptides were loaded to the equilibrated columns under an aqueous acidic condition (0.1% TFA) and desalted by washing column three times with acidified water at low-speed centrifugation, and bound peptides were eluted with 0.1% TFA and 50% acetonitrile. Eluted peptides were dried and reconstituted in a 3% ACN and 0.1% FA in water at a final concentration of 1 μg/μL, and 1 μL was used for LC-MS/MS analysis. Phosphopeptide enrichment was performed with the PolyMac phosphopeptide enrichment kit (Tymora Analytical) following the manufacturer’s recommendation.

### Liquid Chromatography-Tandem Mass Spectrometry Analysis

LC-MS/MS analyses were performed using a Dionex UltiMate 3000 RSLCnano System (Thermo Fisher Scientific, Waltham, MA) coupled online to a Q Exactive High Field Hybrid Quadrupole Orbitrap mass spectrometer (Thermo Fisher Scientific). Peptides were separated in the analytical column at a flow rate of 300 nL/min using a 130 min gradient method using a linear gradient of 2–27% of solvent B in 80 min, 27–40% of B in next 15 min, 40–100% of B in next 10 min at which point the gradient was held at 100% of B for 10 min before reverting back to 2% of B, and holding at 2% of B for 10 min for column equilibration. Solvent A was 0.1% FA in water, and solvent B was 80% acetonitrile and 0.1% FA. After each sample run, the column was washed and equilibrated by using three 30 min LC gradient wash method before injecting the next sample. All data were acquired in data-dependent acquisition (DDA) with a cycle time of 3s/scan and HCD fragmentation. The mass scan ranges were set at 350 to 1600 *m*/*z,* and MS1 and MS2 resolutions were at 120,000 and 15,000 at 200 *m*/*z*, respectively. The automatic gain control (AGC) target was set at 4 × 10^5^, a maximum injection time of 60 ms, a dynamic exclusion of 30s, and an intensity threshold of 5.0 × 10^4^. MS data were acquired in data-dependent mode with a cycle time of 3s/scan.

LC-MS/MS data were analyzed using MaxQuant software (version 1.6.3.3)^42,43^ against canine (*Canis lupus*) database downloaded from UniProt (www.uniprot.org). All searches were carried out with a precursor mass tolerance of 10 ppm; a fragment mass tolerance of 20 ppm, enzyme specificity of trypsin/Lys-C enzyme allowing up to two missed cleavages; and oxidation (methionine) (+15.9949 Da), acetylation protein N-terminus (+42.0106 Da), as variable modifications, and ethanolyl (Cys) (+44.026215 Da) as a fixed modification. Peptides with a minimum of six amino acid residues in length were considered for analysis. The false discovery rate (FDR) of peptide spectral match (PSM) and protein identification was set to 0.01 during the MaxQuant search. The unique plus razor peptides (nonredundant, nonunique peptides assigned to the protein group with most other peptides^44^) were used for peptide quantitation. LFQ (label-free quantitation) intensity values were used for relative protein abundance measurements. Only proteins detected with at least one peptide, ≥2 MS/MS counts, and nonzero LFQ intensities were considered as valid identification and used for bioinformatic analysis.

### Protein Quantification and Statistical Analysis

The Perseus software package (version 2.1.3.0)^45^ was employed to perform downstream bioinformatic and statistical analyses of the MaxQuant output data. Label-free quantification (LFQ) intensities of the identified proteins were first log2-transformed to normalize the distribution. To ensure data quality, proteins annotated as "contaminants", "reverse" (decoy sequences), or "only identified by site" were excluded from the data set. For robust quantitative analysis, only proteins that had valid LFQ intensity values in at least 50% of samples within at least one experimental group were retained for further analysis. For phosphoproteomic data, phosphosite identifications were filtered to include only those with a localization probability ≥ 0.75 (class I phosphosites), thereby ensuring high confidence in site assignment. The total site-specific intensity of phosphorylated residues was then used for downstream analysis.

To interpret the biological relevance of the proteomic and phosphoproteomic changes, pathway and functional enrichment analyses were conducted using the Metascape^46^ and ShinnyGO^47^ platforms. These tools facilitated the identification of enriched biological pathways, molecular functions, and cellular processes associated with the differentially expressed proteins.

## Results

### Study Subjects

Proteomic analysis was performed on the 42 tumor samples obtained from 14 pet dogs in the vemurafenib trial. There were five male and nine female dogs across multiple breeds, with a median age of 11.4 years (range 8–15 years). With the goal to study mechanisms driving relapse after an initial good treatment response, all dogs in this report initially had partial remission (PR) or stable disease (SD) after vemurafenib treatment and later experienced cancer progression (PD). The median progression-free survival with vemurafenib treatment was 220 days (range, 55–608 days). Digested clean peptides from InvUC tumors were analyzed by LC-MS/MS using a data-dependent acquisition (DDA) strategy (Figure 1A).

### Vemurafenib Resistance Accompanied by Metabolic Reprogramming and Upregulation of Proteins That Regulate Cell Proliferation

Tumor heterogeneity as well as complexity of sample preparation and LC-MS/MS analysis workflow can lead to significant proteome variations. Therefore, to distinguish the variation arising from tumor heterogeneity from the variation introduced by the analytical workflow, we performed LC-MS/MS analysis of Pierce Hela Protein Digest Standards (Thermo Fisher Scientific) and identified more than 85% of the peptides and 92% of the proteins common to all three technical replicates, demonstrating good LC-MS/MS reproducibility. The average coefficient of variation (CV) of MS1 intensity (LFQ) of all common peptides was ∼15%, and for proteins, it was ∼10% (data not shown), further ensuring good LC-MS/MS reproducibility. At 1% FDR for both PSM (peptide spectral match) and proteins, we identified 4087 protein groups in total prior to filtering the data. After applying our stringent filtering criteria, we obtained a total of 1544 protein groups (Table S1). This comprehensive canine InvUC tumor proteome data set is summarized in Figure S1. The protein intensities of all quantified proteins spanning up to 3 orders of magnitude were ranked from highest to lowest intensity (Figure S2A). Hemoglobin subunit beta (HBB), histone H2A type 1-A (HIST1H2AA), actin (ACTG1), and histone H2B type W-T (H2BFWT) were among the most abundant proteins. Serine/threonine-protein kinase MRCK beta (CDC42BPB), tyrosine-protein kinase CSK (CSK), and protein phosphatase 1 regulatory subunit 12A (PPP1R12A) were the least abundant proteins.

The differences in global proteomic findings between the three time points, Vem-Pre, Vem-1-month, and Vem-PD, were especially apparent in the dogs that had PR followed by PD (Figure S1) with a reduction in the abundance of most proteins at Vem-1-month and increase in the abundance of these at Vem-PD. Therefore, a subset analysis was performed on samples from these seven dogs that had PR. Overall, results from all seven samples showed consistency in the distribution of LFQ intensity values of protein groups, depicted as violin plots after Log_2_ transformation of the LFQ values (Figure S2B). One-way ANOVA analysis among the three different time points in which samples were collected (Pre-Vem, Vem-1-month, and Vem-PD) resulted in the identification of 128 proteins that were significantly different (ANOVA *p-*value <0.05) and that were used for downstream analysis. Hierarchical clustering of these 128 significant proteins into a heatmap grouped them into four distinct clusters based on their average regulatory profiles across time points (Figure 1B). As illustrated, in two of the clusters, most proteins had a decrease in abundance at Vem-1-month. Interestingly, their levels rebounded at the Vem-PD stage, returning to values comparable to or higher than those observed in Pre-Vem. The two clusters at the top of the heatmap in Figure 1B predominantly contained proteins that were downregulated in Vem-1-month compared to Pre-Vem but exhibited a subsequent increase in abundance by the Vem-PD stage. In contrast, the bottom two clusters consist of proteins that initially had increased abundance at Vem-1-month, which later decreased at Vem-PD. These protein patterns suggest that distinct subsets of proteins could be temporally regulated in response to vemurafenib treatment, potentially indicating stage-specific tumor responses to the treatment. To contextualize the pathways represented by proteins upregulated between Vem-1-month and Vem-PD, we performed gene enrichment analysis using the Reactome database available through the Metascape platform^48^ (Figure 1C). These proteins are primarily involved in metabolic pathways, such as "metabolism of amino acids and derivatives", "selenoamino acid metabolism", and "fatty acyl-CoA biosynthesis". These results are consistent with other findings that disruptions in metabolic pathways are among the most prevalent and easily identifiable characteristics of cancer.^49^ Our results suggest that indeed, a metabolic shift could be an important underlying mechanism of acquired resistance to vemurafenib.

Additionally, the Reactome pathway "chromosome maintenance" was enriched. Proteins mapped to this term are highlighted in Figure 1D. Notable proteins include PCNA, which is involved in DNA replication, repair, and cell cycle progression;^50^ RBBP7 (RB binding protein 7), implicated in regulating cell survival and glycolysis via the activation of the PI3K/AKT pathway;^51^ and SSRP1, which controls cancer cell migration and invasion processes via the activation of the MAPK pathway.^52^ Interestingly, in our previously published study, we observed changes in the RNA levels of several MAPK genes in response to vemurafenib treatment.^26^ The expression of ERK, for example, decreased at Vem-1-month, followed by an increase at Vem-PD.^26^ Consistent with the observation that upregulation of these proteins in cancer could also activate downstream signaling cascades, the "signaling by Rho GTPases" pathway was also found to be enriched in our data set (Figure 1C). Protein trajectories based on LFQ intensities (Figure 2A) show that most dogs exhibited an early decrease in protein abundance during treatment, followed by rebound to baseline or higher levels at the progressive disease (PD) stage. Consistent with this, the violin plots (Figure 2B) display decreased protein abundance during the initial treatment phase with recovery at PD. The magnitude of these changes varied across individual dogs, highlighting interdog heterogeneity in proteomic response. The early decrease of protein LFQ values was particularly notably in Dogs 1, 2, and 4, whereas Dog 3 showed relatively stable expression and Dogs 6 and 7 displayed a modest increase at 1 month that continued to increase slightly at PD.

**Figure 2:**
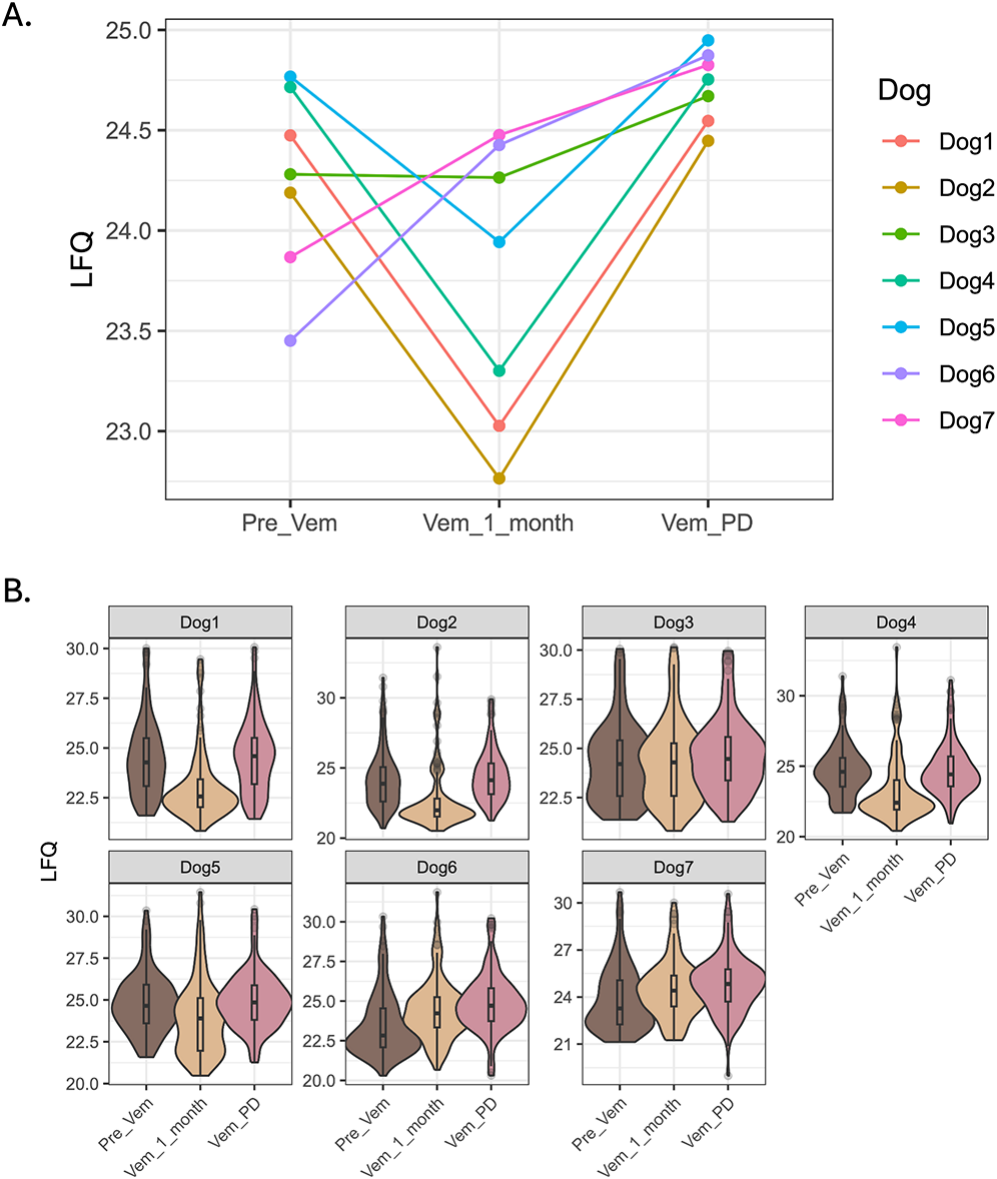
Longitudinal changes in protein abundance across vemurafenib treatment in individual dogs. (A) Line plot showing average LFQ intensity values of significantly different proteins (*p* < 0.05, *n* = 7) for each dog measured at three treatment stages. (B) Violin plots illustrating the distribution of LFQ intensity values of significant protein (*p* < 0.05, *n* = 7) for each dog at the three time points. Embedded boxplots indicate the median and interquartile range. Pre-Vem: Pretreatment, Vem-1-month: after 1 month of vemurafenib treatment, and Vem-PD: at progressive disease. Most dogs exhibit a transient decrease in protein abundances at Vem-1-month, followed by recovery or elevation at Vem-PD, although the magnitude and direction of change vary across individuals.

Because "signaling by Rho GTPase" emerged as a significantly enriched pathway, we examined corresponding changes in LFQ intensity of this pathway-associated proteins, summarized as box plots in Figure 3. Notably, the mitotic checkpoint protein BUB3 showed decreased abundance 1 month after vemurafenib treatment, but its levels returned to baseline at the PD stage. Elevated BUB3 expression has been reported in several cancers and is associated with chromosomal instability, poor prognosis, and resistance to chemotherapy.^53,54^ Similarly, the expression of DYNC1I2 (dynein cytoplasmic 1 intermediate chain 2) was also decreased on the Vem-1-month time point and increased at Vem-PD (Figure 3). Although less studied than other dynein subunits, DYNC1I2 is known to play a role in mitotic spindle organization and chromosome segregation, and thus, its dysregulation could contribute to chromosomal instability, a hallmark of cancer.^55,56^

**Figure 3:**
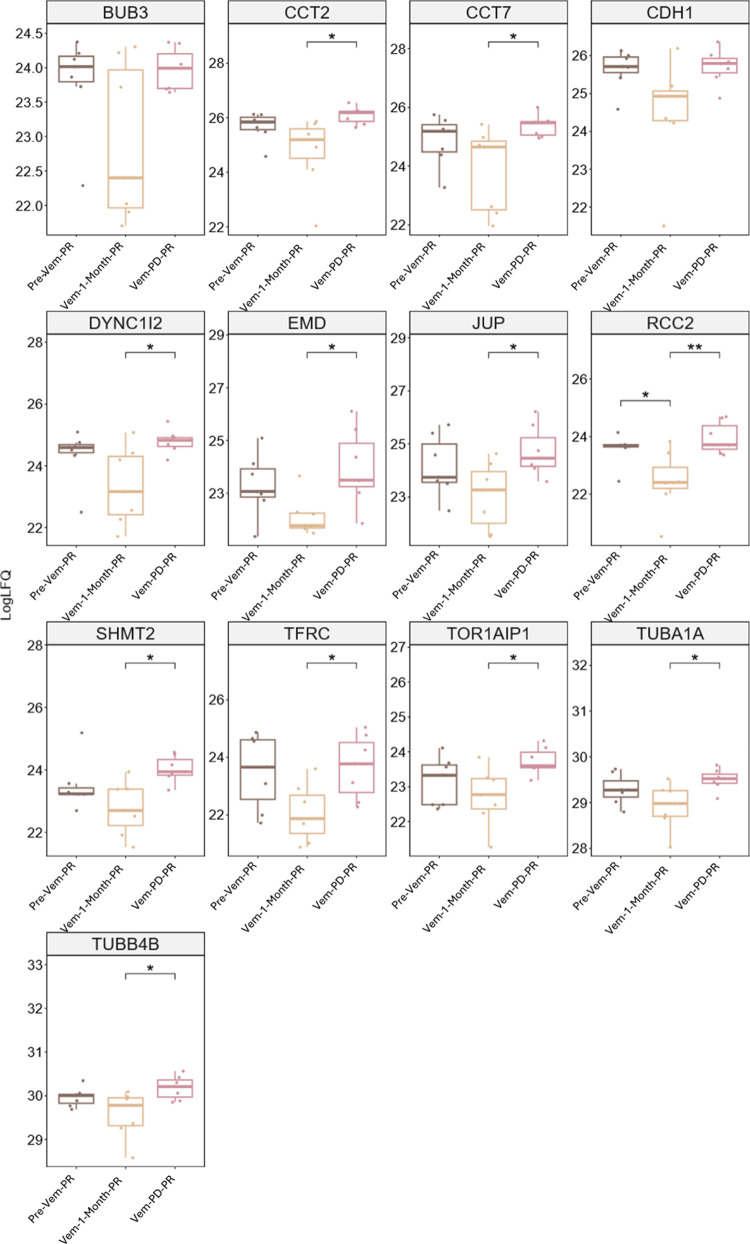
Box plots for proteins mapped to the Reactome term "signaling by Rho GTPases" (∗*p* < 0.05, ∗∗*p* < 0.01; one-way ANOVA followed by Tukey HSD test, *n* = 7). The three time points, Pre-Vem, Vem-1-month, and Vem-PD are presented for dogs that had an initial response of PR.

Gene Ontology Biological Process enrichment analysis, conducted within the ShinnyGO platform,^47^ also underscored the involvement of DNA maintenance and regulatory processes, including "positive regulation of telomerase RNA localization to Cajal body", "positive regulation of protein localization to telomere", and "positive regulation of protein localization to Cajal body" (Figure S3A). These pathways share the involvement of CCT proteins, namely CCT4, CCT7, CCT8, CCT3, and CCT2. The CCT proteins form a TRiC complex that is necessary for telomerase trafficking and proper telomerase function via the proper folding of the TCAB1 protein.^57^ Thus, an increase in telomerase function could be a contributing mechanism involved in vemurafenib resistance.

Subcellular distribution analysis revealed that proteins upregulated in Vem-PD relative to Vem-1-month were primarily localized to the nucleus, proteasome complex, cytoskeleton, and extracellular space (Figure S3B). Notably, many of the upregulated proteins are associated with Cajal bodies, dynamic nuclear substructures involved in snRNP biogenesis, mRNA processing, and the regulation of telomerase activity. Aberrant splicing and dysregulation of Cajal body components, such as coilin, could affect the expression and regulation of tumor suppressors or oncogenes.^58,59^ Although the role of Cajal body proteins in cancer, particularly in urothelial carcinoma, remains unexplored, their effects are emerging as promising areas for research. The upregulation of many Cajal body-associated proteins in InvUC tissues, particularly during vemurafenib treatment, underscores their potential importance and warrants future investigation. Additionally, the tumor suppressor promyelocytic leukemia (PML) protein, a key component of PML nuclear bodies (PML/NBs) involved in senescence, apoptosis, and DNA-damage response,^60,61^ was also detected in 80% of InvUC tissues with variable abundance across samples and vemurafenib treatment time points (Table S1). Despite reasonable PML abundance in InvUC tissues as determined by MS/MS data, its levels did not significantly change over time and showed no correlation with tumor subtypes, treatment progression, or vemurafenib response, suggesting possible functional inactivity. However, the frequent identification of PML in these tumors may implicate its potential involvement in InvUC, underscoring the need for further studies.

### Phosphorylation Response to Vemurafenib Treatment in InvUC

We identified 1093 class I phosphopeptides (peptides with phosphorylation probability ≥ 0.75), which were mapped to 500 phosphoproteins (Table S2). Of those phosphosites, 89.4% were serine, 9.4% were threonine, and 1.2% were tyrosine (Table S2). Similarly to the global proteomic analysis described above, we performed statistical analysis of all class I phosphosites using one-way ANOVA to identify significantly different phosphosites (ANOVA *p*-value <0.1) between the treatment time points in dogs that had PR with vemurafenib. We identified a total of 63 phosphosites significantly different, from which 53 phosphosites were upregulated in Vem-PD relative to Vem-1-month, while only seven phosphosites were downregulated (Figure 4A). Pathway analysis of upregulated phosphoproteins against the Reactome database revealed two enriched pathways, "mRNA splicing – major pathway" and "signaling by Rho GTPases" (Figure 4B). Three proteins were mapped under the term "signaling by Rho GTPases": CPD, CTNNA, and TJP2 (Figure 4C). Interestingly, this pathway was also enriched at the global proteomic data set, indicating a potential reactivation of the Rho GTPase pathways at both total protein as well as phosphoprotein levels that contributes to vemurafenib resistance.

**Figure 4:**
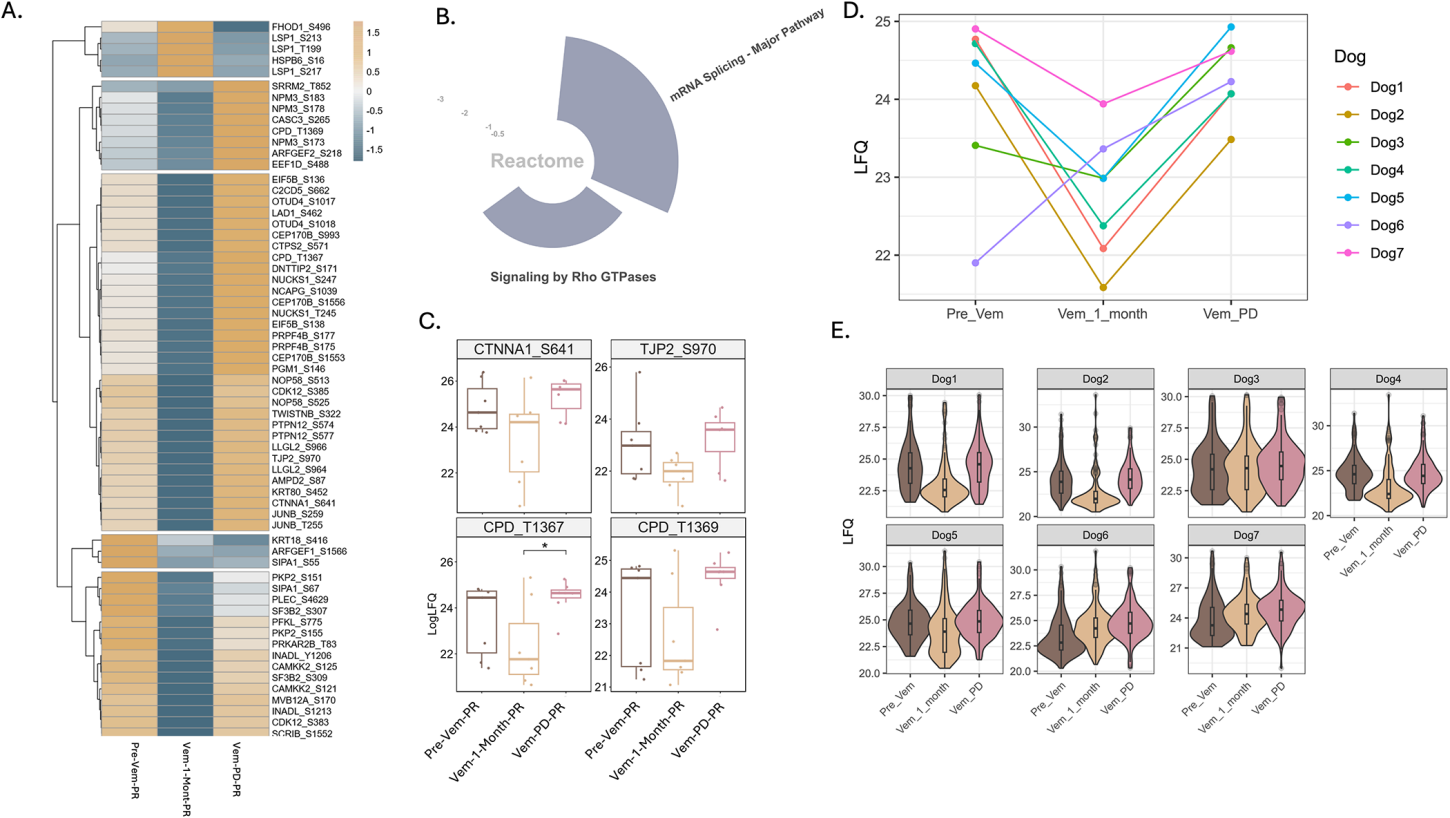
Phosphoproteomics analysis of dogs that had PR after 1-month of vemurafenib treatment followed by cancer progression. (A) Heatmap representation of significantly different phosphosites among the three different time points (Pre-Vem, Vem-1-month, and Vem-PD) (ANOVA *p-*value <0.1). Yellow hue indicates a positive *z*-score values, while blue hue indicates negative *z*-score values. Breaks in the heatmap represent the division between different clusters. (B) Reactome analysis of phosphoproteins in which phosphosites were significantly upregulated in Vem-PD samples compared to Vem-1-month samples. Bar height indicates enrichment – log­(*P*-value). (C) Box plots for phosphosites from proteins mapped to the Reactome term "signaling by Rho GTPases" (*p < 0.05; one-way ANOVA followed by Tukey HSD test, *n* = 7). (D) Line plot showing average LFQ intensity values of significantly different phosphosites (*p* < 0.05) for each dog measured at three treatment stages. (E) Violin plots illustrating the distribution of LFQ intensity values of significant protein (*p* < 0.05) for each dog at the three time points. Embedded boxplots indicate the median and interquartile range. Most dogs exhibit a transient decrease in protein abundances at Vem-1-month, followed by recovery or elevation at Vem-PD, although the magnitude vary across individuals.

Phosphorylation trajectories largely mirrored protein abundance patterns, with most dogs showing an early decline after treatment followed by recovery at the PD stage (Figure 4D,E). The extent of change varied across individual dogs, where Dogs 1, 2, and 4 again showed the greatest decreases of phosphorylation at Vem-1-month, while Dog 6 showed an early increase that was further increased at PD. We also observed three different kinases that were differentially regulated in Vem-PD samples: CAMKK2, CDK12, and PRPF4B (Figure 5A). Each kinase had a significant alteration in two of its phosphosites (Figure 5B), and consistent with our other observations, these kinases control key pathways involved in cancer cell survival and proliferation.

**Figure 5:**
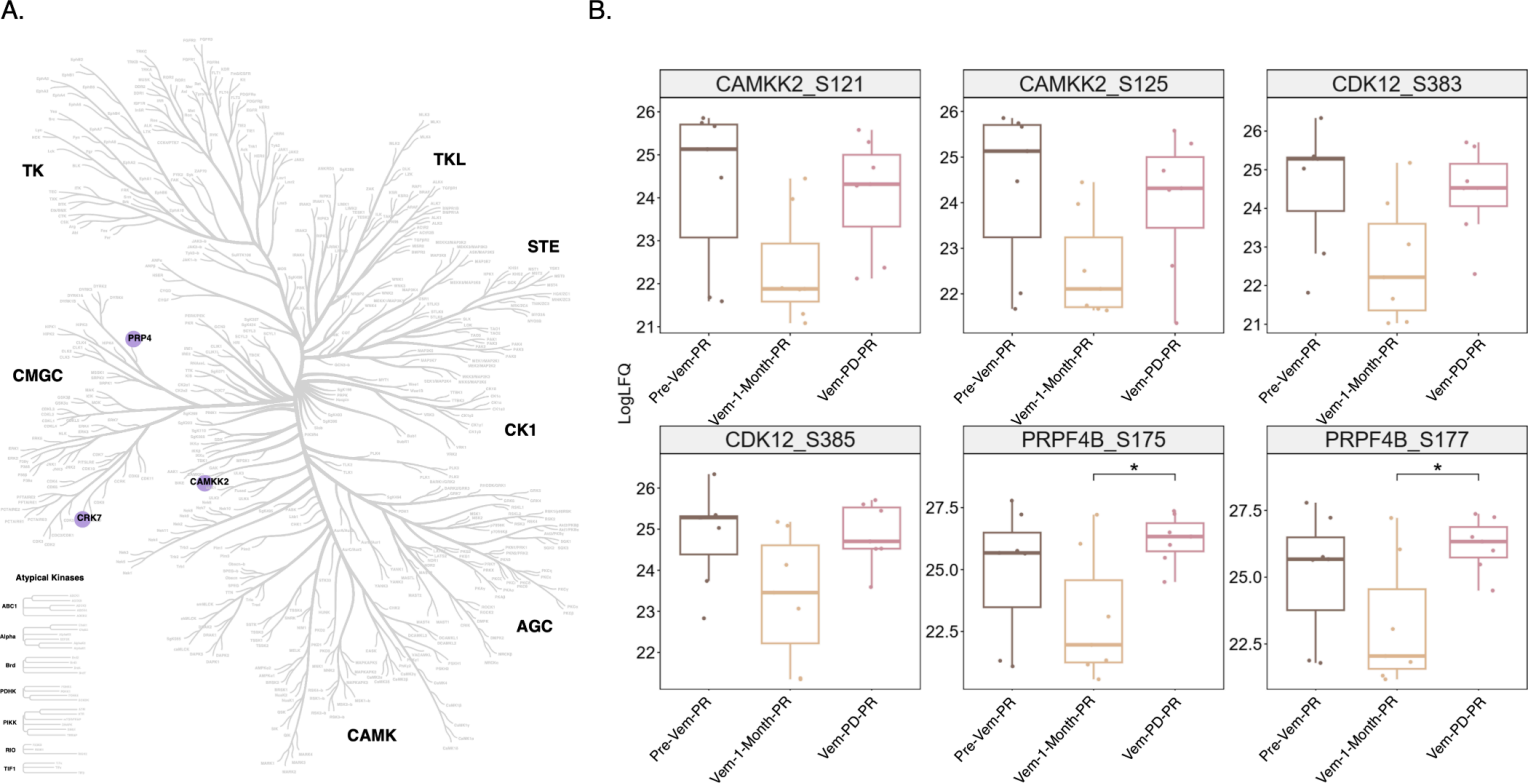
Changes in the kinome of dogs that had PR, with data from all 3 time points included (Pre-Vem, Vem-1-month, Vem-PD). (A) Kinase tree representation of kinases that had significant changes in their phosphorylation status at Vem-PD relative to Vem-1-month (ANOVA *p-*value <0.1). (B) Box plots for the phosphosites for each of the kinases depicted in Figure 4A. (*p < 0.05; one-way ANOVA followed by Tukey HSD test, *n* = 7).

### Protein Signatures Correlated with Initial Sensitivity to Vemurafenib Treatment

Recognizing that the response at 1 month into vemurafenib treatment varies from dog to dog, additional analyses were performed to decipher differences in protein abundances in samples collected at Vem-1-month between dogs that had SD vs dogs that had PR, to better understand the potential mechanisms underlying vemurafenib sensitivity. We identified 154 proteins differentially abundant (*p*-value ≤ 0.05) at Vem-1-month between dogs with SD and dogs with PR (Figure 6A). As shown in the heatmap, these proteins were divided into two clusters: proteins upregulated in dogs with SD containing 123 proteins and downregulated in dogs with SD containing 31 proteins.

**Figure 6:**
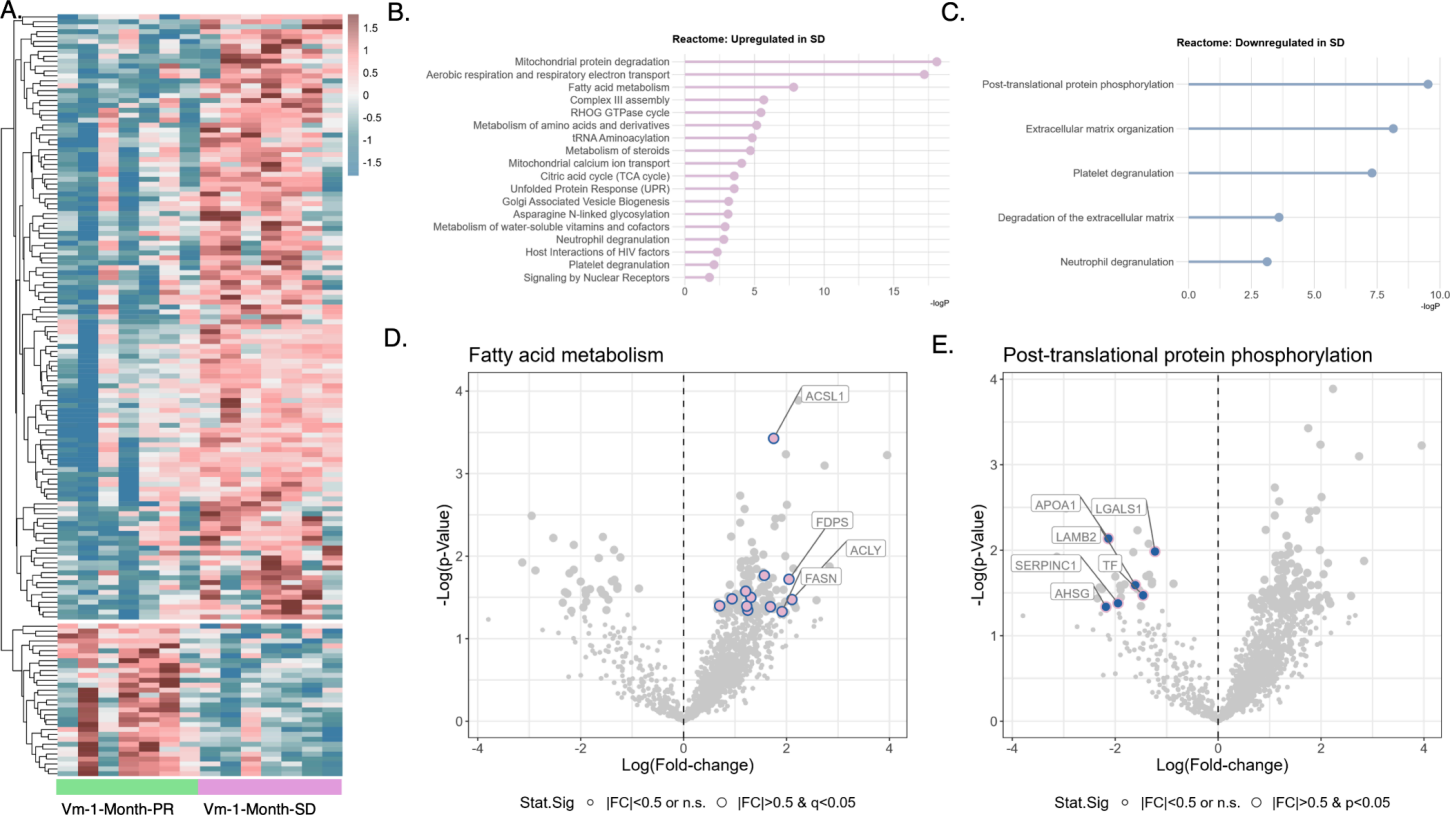
Comparison of global proteomics analysis of dogs with PR versus SD in samples collected at 1 month of treatment (Vem-1-month). (A) Heatmap representation of proteins that were significantly different between dogs with SD vs PD in Vem-1-month samples (ANOVA *p*-value <0.05, *n* = 7). Red hue indicates positive *z*-score values, while blue hue indicates negative *z*-score values. Breaks in the heatmap represent the division between different clusters. (B) Reactome analysis of proteins significantly upregulated in Vem-1-month samples in dogs with SD (Vem-1-month-SD) relative to dogs with PR (Vem-1-month-PR). Bar indicates enrichment – log­(*P*-value). (C) Reactome analysis of proteins significantly downregulated in Vem-1-month-SD relative to Vem-1-month-PR. Bar indicates enrichment −log­(*P*-value). D. Volcano plot highlighting proteins mapped to the Reactome term "fatty acid metabolism". (E) Volcano plot highlighting proteins mapped to "post-translational protein phosphorylation".

The proteins upregulated at Vem-1-month in dogs that had SD were primarily involved in metabolic pathways, mapped to Reactome pathways such as "aerobic respiration and respiratory electron transport", "metabolism of amino acids and derivatives", "metabolism of steroids", and "fatty acid metabolism" (Figure 6B). FASN, one of the proteins mapped to the "fatty acid metabolism" pathway, has been gaining attention as a potential target for anticancer therapies.^62^ FASN could act as an important modulator of not only in lipid metabolism but also glycolysis and amino acid metabolism and is often overexpressed in various cancers.^62,63^ FASN was significantly upregulated in dogs with SD, with a 3.76-fold increase in the protein levels (Figure 6C), indicating lipid metabolism and metabolic reprograming could be important factor contributing to the response to vemurafenib. Gene Ontology Cellular Component analysis also shows that majority of the proteins that were upregulated in dogs with SD were localized to the mitochondria and respiratory chain complexes (Figure S4A), again supporting our observations of metabolic rewiring of tumors with resistance to vemurafenib.

Among the upregulated proteins, several were also mapped to signaling pathways, including "RHOG GTPase cycle" (Figure 6B), "Hallmark Myc targets", and "Hallmark MTORC1 signaling" pathways (Figure S4B). We interrogated the data to determine differences in proteins upregulated in the Vem-1-month samples between dogs with SD vs dogs with PD. We found that 38 proteins were commonly upregulated in dogs with SD compared to dogs with PR (Figure S5A,B). Interestingly, these proteins were mapped to several metabolic, signaling, and cell cycle related pathways, including "fatty acyl-CoA biosynthesis", "RHOQ GTPase cycle", "RHOG GTPase cycle", and "mitotic metaphase and anaphase" (Figure S5C). These observations could indicate potential overlap in molecular mechanisms at play in dogs with the less-than-optimal response at Vem-1-month, i.e., SD rather than PR, and mechanisms in cancer progression, i.e., found at Vem-PD. Conversely, few proteins were downregulated in dogs with SD vs dogs with PR, and these proteins were primarily involved in phosphorylation pathways (Figure 6D,E).

### Functional Analysis of Activated Cellular Pathways Revealed by Differential Phosphoproteomics

Consistent with global data, our phosphoproteomics data reveals 79 significantly changing phosphosites (*p*-value ≤ 0.1), of which the majority (64 phosphosites) were upregulated at Vem-1-month in dogs with SD vs dogs with PR (Figure 7A). In agreement with our previous observations, these phosphorylated proteins were primarily involved in signaling and metabolic pathways (Figure 7B), notably "signaling by Rho GTPases" and "glycerophospholipid biosynthesis" (Figure 7C,D), reinforcing the involvement and importance of these pathways in vemurafenib response. One of the major phosphoproteins with reduced phosphorylation in dogs with SD vs dogs with PR was LSP1 (lymphocyte-specific protein 1), which was phosphorylated at S201, S213, S217, and T199 (Figure 7A). This protein is involved in cytoskeleton dynamics and cell migration, and its phosphorylation could be involved in these processes. While the specific role of LSP1 phosphorylation in InvUC is unknown, differential phosphorylation of LSP1 could influence InvUC tumor heterogeneity and tumor response to vemurafenib treatment. Additionally, phosphorylation of CTNNA1 (catenin alpha-1) and PRKAR2A (protein kinase cAMP-dependent type II regulatory subunit alpha), key signaling proteins, were also decreased in dogs with SD compared to dogs with PR (Figure 7A), and this could also be relevant to tumor progression and vemurafenib treatment responses.

**Figure 7:**
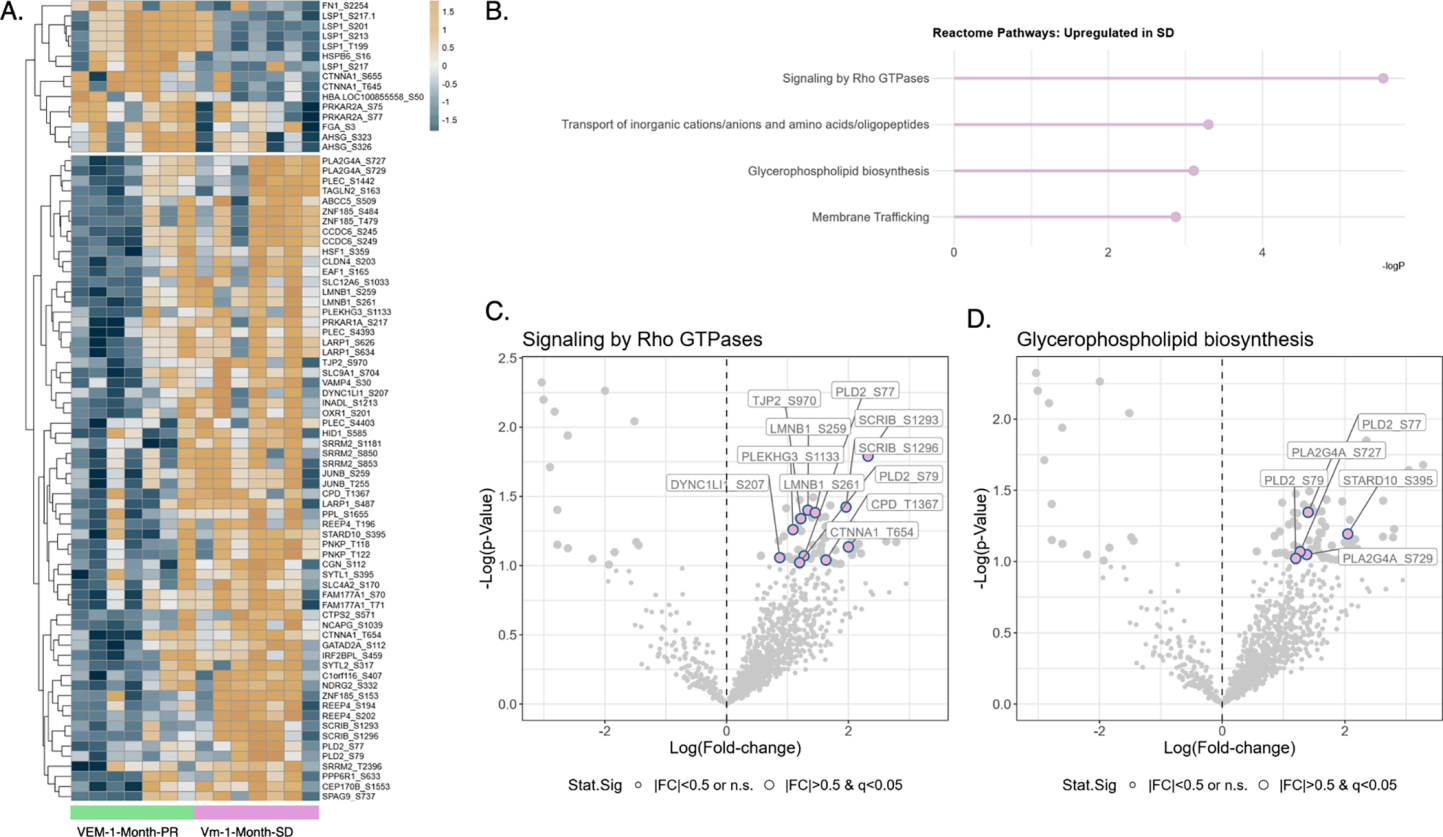
Results of phosphoproteomics analysis of dogs with PR versus SD at 1 month into vemurafenib treatment. (A) Heatmap representation of phosphosites that were significantly different at the 1 month time point between dogs with SD (Vem-1-month-SD) compared with dogs with PR (Vem-1-month-PR) (ANOVA p-value <0.1, *n* = 7). Yellow hue indicates positive *z*-score values, while blue hue indicates negative *z*-score values. Breaks in the heatmap represent the division between different clusters. (B) Reactome analysis of significantly upregulated phosphosites in Vem-1-month-SD relative to Vem-1-month-PR. Bar indicates enrichment −log­(*P*-value). (C) Volcano plot highlighting phosphosites of proteins mapped to the Reactome term "signaling by Rho GTPases". (D) Volcano plot highlighting phosphosites of proteins mapped to the "glycerophospholipid biosynthesis".

In contrast, Vem-1-month samples from dogs with SD had increased phosphorylation of several proteins when compared to samples from dogs with PR (Figure 7A). Notably, this included enhanced phosphorylation of key regulatory proteins such as LMNB1 (involved in nuclear structure and organization), SRRM2 (a splicing factor linked to RNA processing), PLA2G4A (a phospholipase involved in lipid signaling), REEP4 (involved in nuclear envelop formation), JUNB (a transcription factor implicated in cell proliferation and stress response), and CCDC (multiple coiled–coiled domain containing proteins). These results point to a diverse and complex, time-dependent phosphorylation landscape in InvUC tissues and underscore the need for more studies to investigate these molecular changes in the context of tumor progression and therapeutic resistance.

## Discussion

InvUC initially responds very favorably to vemurafenib in dogs, as is observed in humans with BRAF-driven melanomas. Favorable immune changes have also been observed with vemurafenib treatment.^37^ This, however, is followed by drug resistance in dogs and humans. Vemurafenib had a 38% remission rate and 6 months of progression-free interval (PFI) in dogs with InvUC, which compared favorably to the more typical 25% remission rate and 4 month PFI with other single-agent therapies in dogs with InvUC.^64,65^ Our earlier transcriptomic analysis of cystoscopic biopsies collected during the phase I/II trial of vemurafenib, before treatment, during the first month, and at a disease progression, revealed early T lymphocyte infiltration during the first month, which was subsequently followed by immune evasion as cancer progressed.^26^ The observed downregulation of several key proteins and phosphosites associated with invasive and drug-resistant phenotypes from this study further align with the initial beneficial effects of vemurafenib in dogs followed by drug resistance, supporting that the InvUC tumor response to vemurafenib in dogs involves an initial antitumor effect, as is the case in humans treated with BRAF-targeted therapy.^32,37,66^ The proteomic characterization of InvUC tissues in this study broadened our knowledge of some of the specific molecular features that can contribute to vemurafenib resistance. This study represents the first of its kind comprehensive proteomic and phosphoproteomic characterization of canine InvUC tissues in the context of vemurafenib therapy and acquired tumor resistance to therapy. The proteomic landscape before and during vemurafenib treatment revealed differentially regulated proteins and pathways that were distinguishable in luminal and basal subtypes, as well as in dogs exhibiting remission or SD. This data set could serve as a clinically relevant proteomic resource for larger scientific investigation, especially as such data are sparse in dogs with InvUC.

One of the important observations in this study was the consistent trend of initial downregulation of proteins and phosphorylation levels in tumors following 1 month of vemurafenib treatment, followed by a rebound of those proteins at the point of cancer progression, both at the total protein and phosphoprotein level (Figures 1–4). This trend suggests that InvUC tissues exhibit an early cancer suppressive response to vemurafenib that is later reversed as tumors adapt and develop resistance. The proteome characterization distinguished pathways including chromosome maintenance, mRNA splicing, signaling by Rho GTPase, telomerase assembly and regulation, protein degradation, and lipid metabolism.

Functional enrichment of upregulated proteins at the Vem-PD stage compared to Vem-1-month samples when the cancer was controlled revealed changes of diverse metabolic pathways including amino acid metabolism, selenoamino acid metabolism, and fatty acyl-CoA biosynthesis, all of which are central to metabolic reprogramming and could have contributed to vemurafenib resistance. These results are consistent with the idea that cancer cells adopt alternative metabolic strategies to survive targeted inhibition of oncogenic signaling pathways such as BRAF-MAPK.^67,68^ Among the notable protein-level changes associated with acquired resistance were those involved in chromosomal maintenance and telomerase regulation (Figure S3A). Proteins such as PCNA, RBBP7, and SSRP1, which are critical to DNA replication and repair, were significantly upregulated at the Vem-PD stage (Figure 1D). These changes were also reflected at the phosphoproteome level, where we observed increased phosphorylation levels of proteins involved in mRNA splicing and Rho GTPase signaling (Figure 4B), pathways linked to proliferative and immune cell migration in cancer. One of the most consistent pathways identified in both proteomic and phosphoproteomic data sets was "signaling by Rho GTPases". This validation via both total proteins and phosphoproteins underscores the functional reactivation of this pathway in resistant tumors. Proteins such as BUB3 and DYNC1I2, which are involved in mitotic checkpoint control and chromosomal segregation, also followed similar expression patterns across time points, further supporting the idea that chromosomal instability and cell progression are key features of the resistant state.^69,70^ Additionally, the upregulated phosphorylation of CPD, CTNNA1, and TJP2 within the Rho GTPase signaling context implies increased cytoskeletal remodeling and potentially enhanced migratory behavior. These findings align with prior observations in human cancers, where reactivation of Rho-mediated signaling confers invasive and therapy-resistant phenotypes.^71,72^ Furthermore, the upregulation of multiple chaperonin-containing TCP1 subunits (CCT2, CCT3, CCT4, CCT7, and CCT8) (Figures 1B and 3) in Vem-PD suggests an increase in telomerase function via improved folding and trafficking of TCAB1.

Another interesting observation in this study was the identification of many nuclear proteins including those involved in nuclear substructures such as Cajal bodies and PML nuclear bodies. While PML was detected in the majority of samples, its levels remained unchanged across treatment time points and did not correlate with BRAF status or tumor subtype. The absence of phosphorylation and the lack of dynamic expression suggest that PML could be functionally inactive in InvUC. However, their consistent presence implies that PML nuclear bodies might still influence the tumor microenvironment or chromatin dynamics in a context-dependent manner. Future studies using IHC or proximity labeling could further elucidate their regulation and interaction networks in drug-resistant tumors. GO enrichment terms related to telomere localization and Cajal body dynamics, which are implicated in snRNP biogenesis and telomerase assembly, could point to a potential mechanism underlying vemurafenib resistance in InvUC via these subnuclear structures. These findings warrant further investigation into the regulation of telomerase and integrity, structure, and functions of nuclear bodies, such as PML nuclear bodies and Cajal bodies, in this cancer type.

Additionally, several significantly altered proteins and phosphoproteins identified between pre- and postvemurafenib treatment, including CDK12, CAMK2, NCAPG, PCNA, FASN, and PSMD14 are well-known therapeutic targets of various cancers. These findings underscore the dynamic proteomic and phosphoproteomic remodeling induced by vemurafenib and highlight the potential of this data set to advance our understanding into therapeutic response and tumor resistance in dogs with InvUC harboring BRAF^V595E^ mutation.

## Conclusions

Cancer remains a leading cause of death worldwide. A combination of genetic and environmental factors, including exposure to cigarette smoke, toxic chemicals, and radiation, is known to cause various cancers including InvUC. In this study, we sought to determine the differences in the total proteome and phosphoproteome profiles of different InvUC tissues before and during BRAF-targeted vemurafenib therapy. The study shed light on the expression of many cell cycle and tumor suppressor proteins and their post-translational regulation pre- and post-vemurafenib treatment. Our data reveal a complex interplay between metabolic reprogramming, telomerase function, chromosomal maintenance, and reactivation of proliferative signaling pathways such as Rho GTPase that could lead tumor resistance to vemurafenib in canine InvUC. The emergence of these resistance features across multiple regulatory layers underscores the need to continue to develop combination therapeutic strategies that simultaneously target metabolic flexibility and nuclear-signaling hubs. Furthermore, the involvement of Cajal body components and TRiC complex proteins introduces a new dimension to the understanding of therapy resistance, highlighting novel candidates for functional and mechanistic studies and potential therapeutic interventions. Future investigations should prioritize validation of these targets using genetic manipulation and functional assays, as well as expansion into spatial and cell-type-specific proteomics or single-cell analyses to dissect tumor heterogeneity at higher resolution. Overall, these findings not only contribute to the growing body of knowledge on BRAF inhibitor resistance but also validate the use of naturally occurring canine tumors as a powerful translational model to study mechanisms of therapeutic resistance in urothelial carcinoma.

## Limitation of the Study

Proteomic and phosphoproteomic analyses have been successfully applied to a range of tumors, including colorectal cancer,^73^ brain tumors,^74^ thyroid cancer,^75^ gastric cancer,^76^ breast cancer,^77,78^ and others,^79^ using human tissues, animal models, and patient-derived xenografts. However, this study is the first to perform a comprehensive proteomic and phosphoproteomic characterization of canine InuUC tumors. While our analysis offers valuable insights into these tumors, we recognize several limitations. The depth of proteome coverage, as well as the number of mapped phosphosites, was relatively modest. We relied on a single protease (trypsin) for digestion; employing multiple proteases, as we have previously demonstrated,^80^ along with protein- or peptide-level fractionation or organelle separations, could substantially improve coverage.

This study included a relatively small number of animals per group. Increasing the sample size would enhance the robustness and statistical power of the analysis, allowing for more confident conclusions to be drawn. In addition, we performed this study using the entire tumor biopsy, which may contain stromal admixture, as well as residual blood, which may have contributed to the detection of proteins abundant in the stromal admixture rather than the tumor cells themselves. Addressing these limitations using advanced mass spectrometry instrumentation and improved sample preparation strategies would further enhance a more comprehensive molecular characterization and deeper understanding of how the tumor microenvironment evolves in different contexts, including before and after targeted vemurafenib therapy and help to more broadly generalize our findings.

## Supplementary Material





## Data Availability

The mass spectrometry proteomics data have been deposited to the ProteomeXchange Consortium via the PRIDE^81^ partner repository with the data set identifier PXD062194. Note that a smaller subset of the phosphoproteomics data set was used in one of our previous publications,^26^ where it was employed to validate some of the kinases identified by RNA-seq analysis. As such, no further exploration of the data set was performed then, and all analyses and conclusions of the present study are completely independent.

## Notes

Figure S1. Heatmap representation of all quantified proteins; Figure S2. Ranked plots and violin plots depicting all quantitated proteins and their intensity; Figure S3. Proteins upregulated across three time points in dogs showing partial remission after treatment; Figure S4. Proteins significantly enriched in 1 month after treatment in dogs showing stable disease versus partial remission; Figure S5. Significant protein overlaps among dogs with different responses pre- and post- treatment (PDF)
Table S1. MaxQuant search results and Perseus analysis of all quantified proteins; Table S2. MaxQuant search results and Perseus analysis of all mapped phosphosites (XLSX)

## ω

Current address: Department of Biochemistry and Molecular Biology, Ferguson College of Agriculture, Oklahoma State University, Stillwater, Oklahoma 74075, United States
