# Supplementary material for: Proteomic Signatures of Vemurafenib Resistance in Canine Urothelial Carcinoma Harboring the BRAFV595E Mutation
Source: J Proteome Res. 2026 Jun 26;25(8):4262–75. doi: 10.1021/acs.jproteome.6c00246 (PMC13459542; doi:10.1021/acs.jproteome.6c00246)
Supplement: Supplementary file 1 [file pr6c00246_si_001.pdf]

**Supporting Information**

# Proteomic signatures of vemurafenib resistance in canine urothelial carcinoma harboring the *BRAF*$^{V595E}$ mutation

Rodrigo Mohallem[1], Deepika Dhawan[2], Alexander W. Enstrom[2], Deborah W. Knapp[2,3,*], and Uma K. Aryal[1,ϖ,*]

[1]Department of Comparative Pathobiology, College of Veterinary Medicine, Purdue University, West Lafayette, IN 47907, USA

[2]Department of Veterinary Clinical Sciences, College of Veterinary Medicine, Purdue University, West Lafayette, IN 47907, USA.

[3]Purdue University Institute for Cancer Research, West Lafayette, IN 47907, USA.

**[ϖ]Current address**

Department of Biochemistry and Molecular Biology, Ferguson College of Agriculture, Oklahoma State University, Stillwater, OK 74075, USA

**[*]Corresponding authors**

Deborah W. Knapp
Department of Veterinary Clinical Sciences, Purdue University
625 Harrison Street
West Lafayette, IN 47907-2026
Phone: 765-494-9900
E-mail: knappd@purdue.edu

Uma K. Aryal
Department of Biochemistry and Molecular Biology
Oklahoma State University
246 Noble Research Center
Stillwater, OK 74075
Phone: 405-744-6206
E-mail: uaryal@okstate.edu

**Table of Contents:**

**Supplementary Figures**

Figure S1. Heatmap representation of all quantified proteins.

Figure S2. Ranked plots and violin plots depicting all quantitated proteins and their intensity.

Figure S3. Proteins upregulated across three time points in dogs showing partial remission after treatment.

Figure S4. Proteins significantly enriched in 1-month after treatment in dogs showing stable disease versus partial remission.

Figure S5. Significant protein overlaps among dogs with different responses pre- and post-treatment.

**Supplementary Tables**

Table S1. MaxQuant search results and Perseus analysis of all quantified proteins.(XLSX)

Table S2. MaxQuant search results and Perseus analysis of all mapped phosphosites.(XLSX)

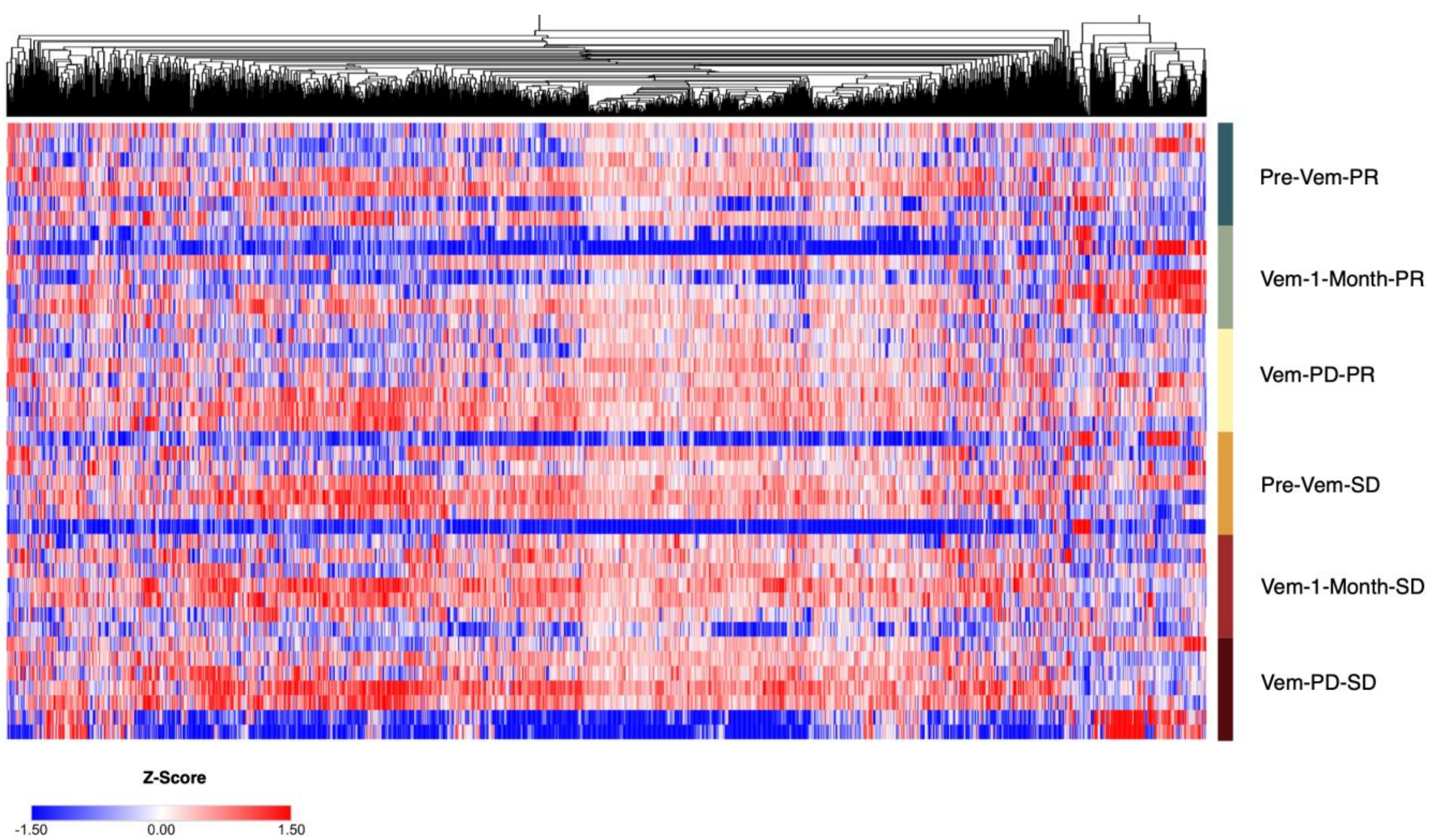


**Figure S1**. Heatmap representation of all quantified proteins in the global proteomics analysis. Red hue indicates positive z-score values, while blue hue indicates negative z-score values. Results are grouped according to results and time point: dogs that had PR with samples

collected before vemurafenib (Pre-Vem-PR) at 1-month into treatment (Vem-1-month-PR), and at cancer progression (Vem-PD-PR); and dogs that had SD with samples from the 3 time points (Pre-Vem-SD, Vem-1-month-SD, and Vem-PD-SD respectively).

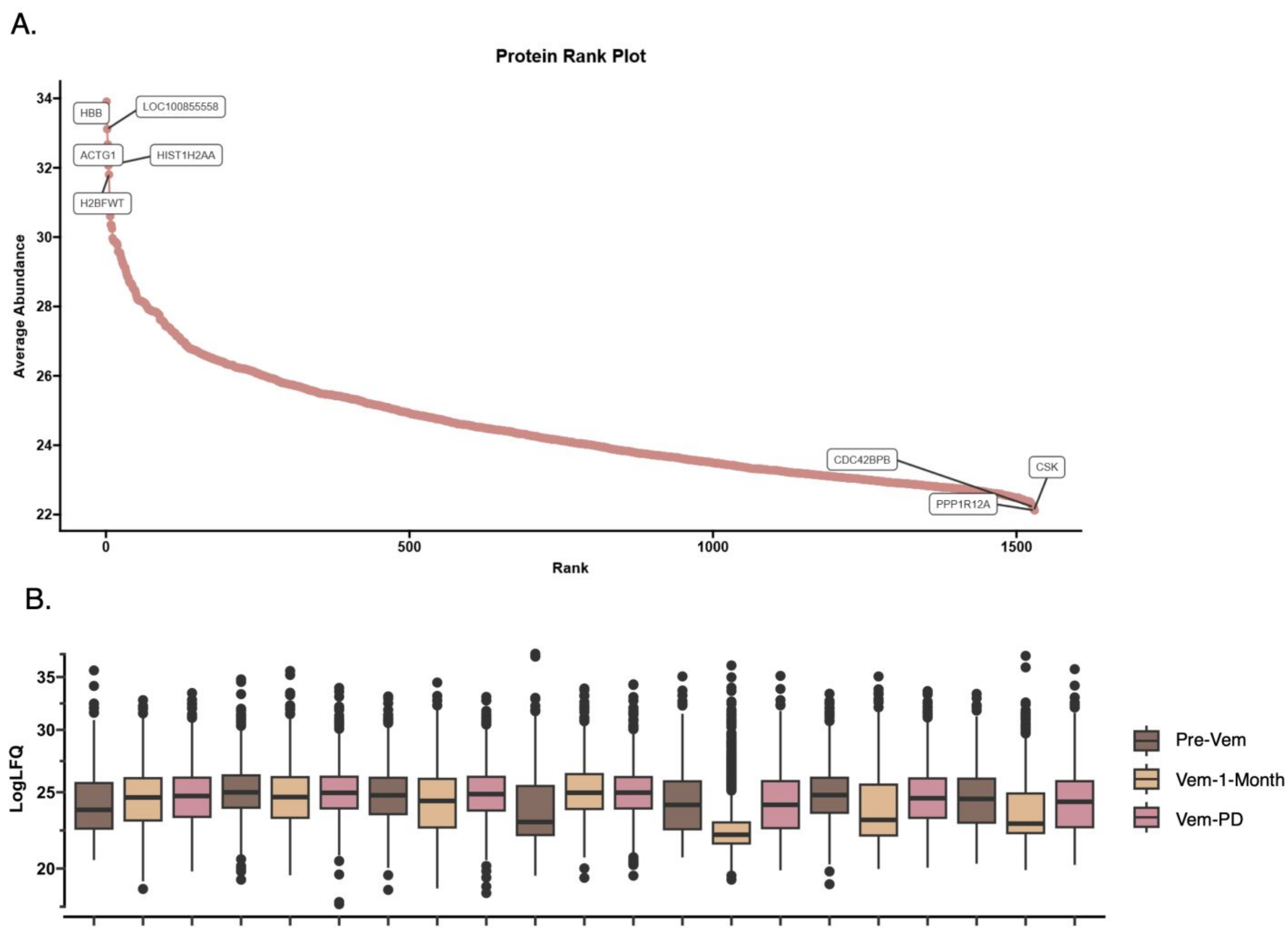


**Figure S2**. Qualitative analysis of global proteomics. **A.** Ranked plots depicting all quantified proteins in global analysis. Lower ranks correspond to proteins with the highest average Log(LFQ) intensity. **B.** Results from dogs that had PR with vemurafenib treatment. Violin plots depicting Log(LFQ) intensity values at 3 time points (Pre-Vem, Vem-1-month, Vem-PD). Colors (see key) indicate timepoints at which samples were collected.

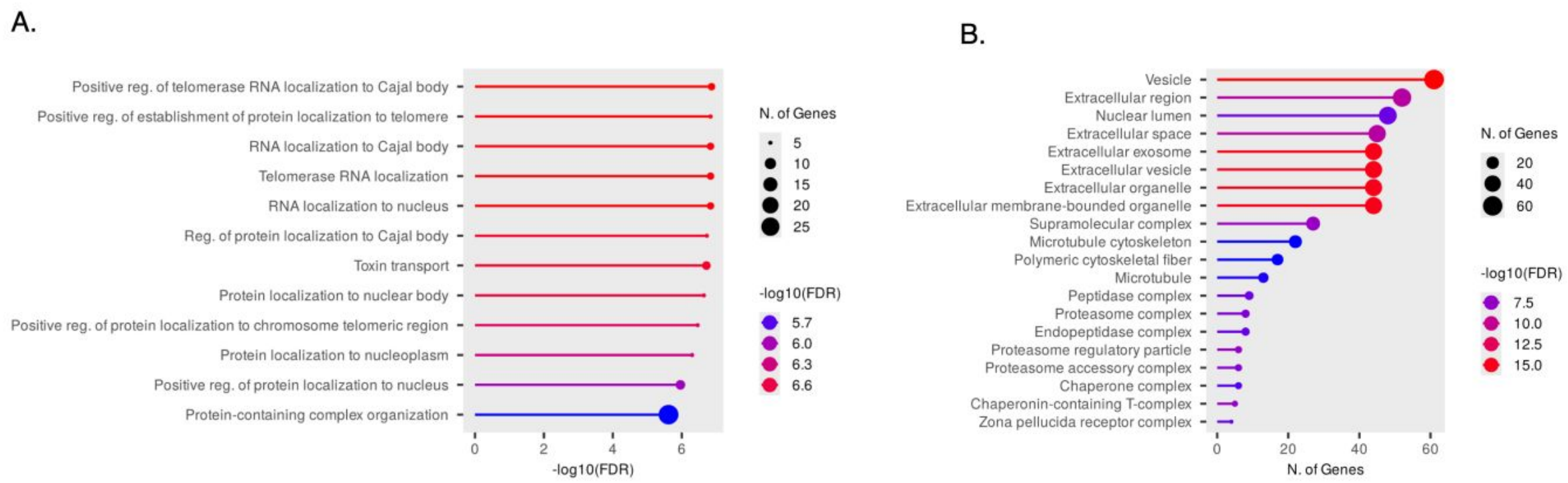


**Figure S3**. Enrichment analysis of proteins significantly upregulated among the three different timepoints in dogs with a best tumor response classified as "PR" upon vemurafenib treatment (ANOVA *p*-value <0.05) **A:** Gene Ontology Biological Processes and **B:** Gene Ontology Cellular Compartment.

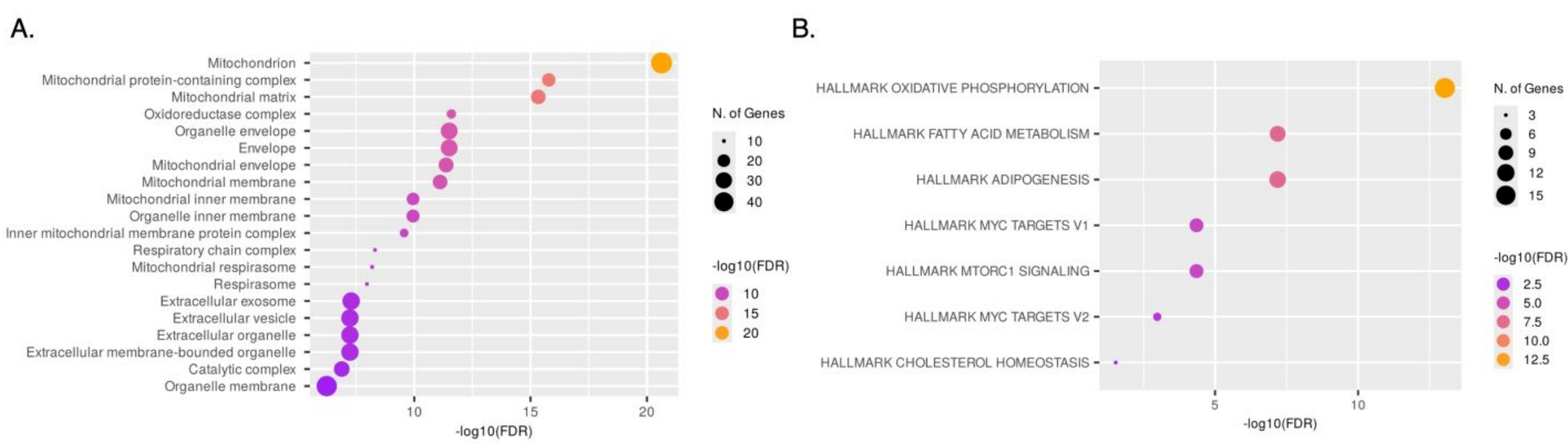


**Figure S4**. Enrichment analysis. Proteins significantly enriched in samples collected at Vem-1-month in dogs with SD vs dogs with PR (ANOVA *p*-value <0.05) **A:** Gene Ontology Cellular Compartment and **B:** Hallmark Molecular Signatures.

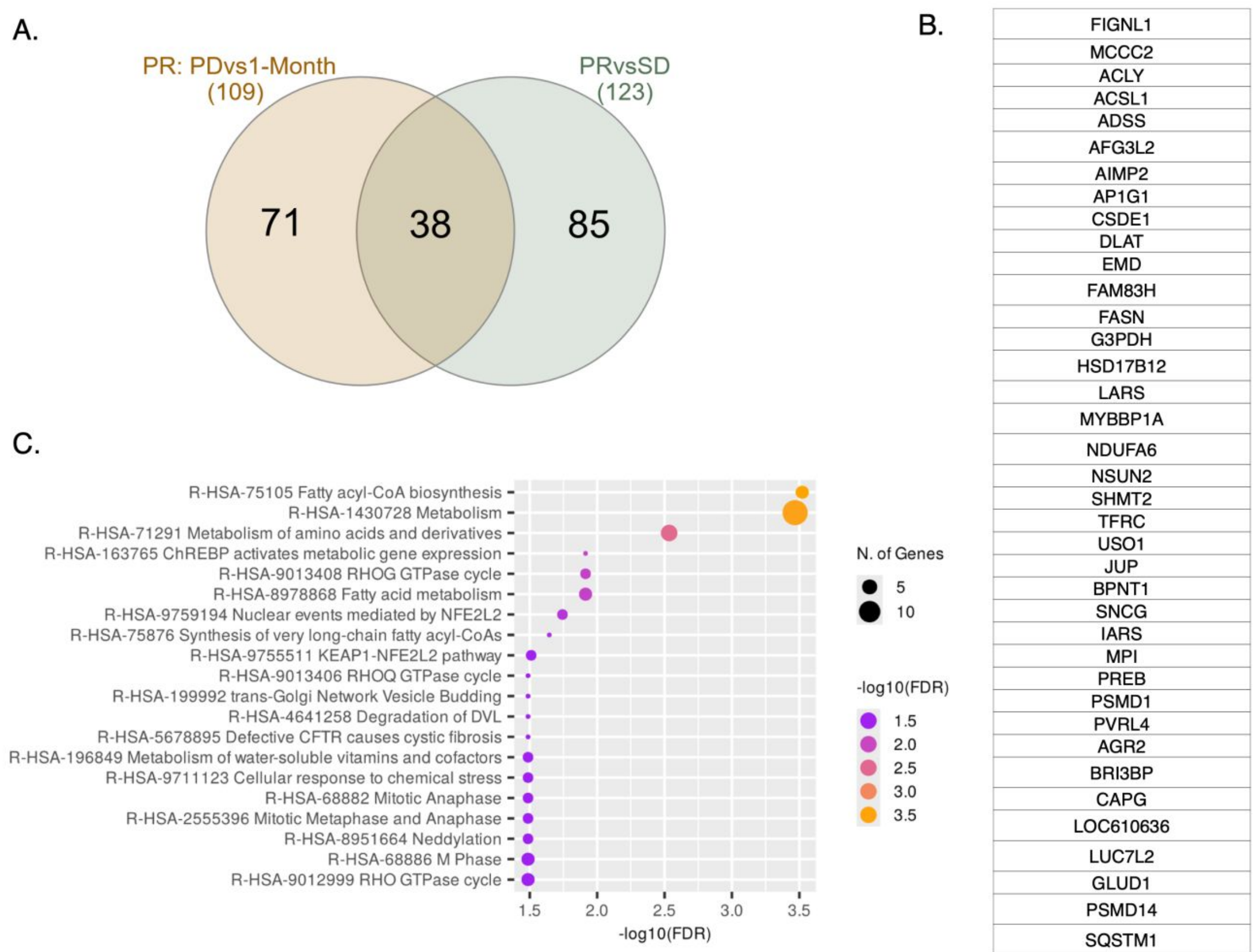


**Figure S5**. Overlap of significantly regulated proteins between the two comparisons with the first comparison being in samples collected from dogs that had PR at the Vem-PD time point (Vem-PD-PR) vs the 1-month time point (Vem-1-month-PR), and the second comparison being in samples collected at Vem-1-month from dogs that had PR vs dogs that had SD. **A.** Venn diagram depicting the common and unique proteins that were significantly upregulated in each comparison. **B.** List of common proteins significantly upregulated in the two comparisons: Vem-PD-PR vs Vem-1-Month-PR, and Vem-1-month-SD vs Vem-1-month-PR. **C.** Reactome pathways enriched for the proteins listed in Supplementary Figure 5B.
